# Superoxide dismutase VPA1514 in *Vibrio parahaemolyticus* protects against environmental stresses

**DOI:** 10.1371/journal.pone.0329351

**Published:** 2025-08-14

**Authors:** Paula Hsu, Hin-chung Wong, Chung-Tao Tang

**Affiliations:** 1 Department of Microbiology, Soochow University, Taipei, Taiwan, Republic of China; 2 School of Medicine, College of Medicine, I-Shou University, Kaohsiung, Taiwan, Republic of China; Nitte University, INDIA

## Abstract

*Vibrio parahaemolyticus* is a foodborne enteropathogen that has become a global concern since the emergence of the pandemic O3:K6 strain in 1996. Three putative superoxide dismutases (SODs), namely FeSOD (VP2118), MnSOD (VP2860) and CuZnSOD (VPA1514), are found in *V. parahaemolyticus*. In this study, the VPA1514 mutant and gene complementary strains of *V. parahaemolyticus* were constructed to investigate the function of VPA1514 against environmental stresses. The growth of the VPA1514 mutant strain in broth medium under sublethal stress of H_2_O_2_ was significantly slowed relative to that of the wild-type strain. The survival rate of this VPA1514 mutant strain, when challenged by lethal acetic acid for two hours, was significantly lower than that of the wild-type strain. Moreover, the presence of the complementary VPA1514 gene significantly ameliorated the survival of this *V. parahaemolyticus* mutant strain. VPA1514 also showed the effect on the survival of a SOD mutant strain of *Escherichia coli* against acetic acid. In summary, this study demonstrated that VPA1514 protects against exogenous H_2_O_2_ and the lethal concentrations of acetic acid in *V. parahaemolyticus*.

## Introduction

*Vibrio parahaemolyticus* is a marine halophilic Gram-negative bacterium and a foodborne pathogen prevalent in Taiwan and other Asian countries. Since the emergence of the pandemic O3:K6 strain in 1996, *Vibrio parahaemolyticus* has also become a significant public health concern in North America and other continents [1–5]. In its natural habitats and in food processing environments, *V. parahaemolyticus* encounters various environmental stresses, which are frequently associated with the accumulation of detrimental reactive oxygen species (ROS) [6–9].

Organic acids, such as acetic acid and lactic acid, are commonly used as condiments, acidulants and food preservatives [10], especially in the preparation of sashimi and seafood, and are common environmental stresses for *V. parahaemolyticus*. Vinegar typically contains 4–8% (about 0.66-1.33M) acetic acid, and soy sauce generally contains 1–2% organic acids, with acetic acid, lactic acid and citric acid being the major components [11,12].

Bacterial resistance to acid stress is often linked to oxidative stress response mechanisms, which include the superoxide dismutases (SODs, EC 1.15.1.1). SODs play a crucial role in neutralizing reactive oxygen species (ROS) generated under acidic conditions [13–16]. For example, lactic acid triggers iron-mediated oxidative stress that can be ameliorated by MnSOD and iron chelators [13]. In some Gram-positive bacteria, defense against acid stress is associated with the cytosolic SODs, such as the MnSOD in *Streptococcus thermophilus* [13], *Lactococcus lactis* [14], and *Staphylococcus aureus* [15]. Also, cytosolic MnSOD and FeSOD are related to the acid resistance of *V. vulnificus* [16]. In contrast, CuZnSOD has not been demonstrated to have a role in the acid resistance of bacteria [17,18].

A search of the genome sequence of *V. parahaemolyticus* RIMD 2210633 (GenBank ID: NP801024) revealed three SOD genes encoding VP2118 (FeSOD), VP2860 (MnSOD) and VPA1514 (CuZnSOD) [19]. Investigation of the knockout mutants of these SOD genes indicated that VP2118 is the chief SOD of *V. parahaemolyticus*, acting against hydrogen peroxide, hypoxanthine-xanthine oxidase or paraquat [20]. Bacterial FeSOD and MnSOD are usually cytosolic proteins with similar functions [21,22], and VP2860 (MnSOD) in *V. parahaemolyticus* may have a compensatory function for VP2118 (FeSOD) [20]. The MnSOD protein of *E. coli* incorrectly incorporated with iron exhibits a catalytic peroxidase-catalase activity [23], whereas the expression and activity of MnSOD/FeSOD are regulated by Ferric uptake regulator (Fur) in *S. enterica* [24]. These studies suggest that the metallic composition of a culture medium could influence the expression and activity of SOD. Therefore, it is reasonable to investigate the expression of MnSOD and homologous enzymes in *V. parahaemolyticus*, *V. mimicus*, and *V. vulnificus* under iron-limiting conditions, as these enzymes have been implicated in oxidative stress resistance against H₂O₂ and KCN [25]. VPA1514 (CuZnSOD) is a periplasmic protein like other homologous CuZnSODs in Gram-negative bacteria [26], and the function of VPA1514 has not been characterized. In this study, we aimed to characterize the protective roles of the putative CuZnSOD (VPA1514) in *V. parahaemolyticus* under environmental stress conditions, including organic acid. We used a gene deletion mutant to assess the functional role of VPA1514 and further validated our findings in a CuZnSOD-deficient *E. coli* strain as a heterologous model system.

## Materials and methods

### Bacterial strains and culture conditions

The bacterial strains and plasmids used in this study are listed in Table 1. *V. parahaemolyticus* strain KX-V231 (Kanagawa phenomenon positive, serotype O3:K6) was stored at −80°C in Microbank cryovials (Pro-Lab Diagnostics, Austin, TX, USA) [27]. *V. parahaemolyticus* was cultured at 37°C on Luria-Bertani Agar (Becton, Dickinson Diagnostic Systems, Sparks, MD, USA) supplemented with 3% sodium chloride (LA-3% NaCl), or in Luria-Bertani Broth with 3% NaCl (LB-3% NaCl). *E. coli* was cultured at 37°C on LA or LB. Chloramphenicol (final concentration, 5 or 20 μg/ml) or ampicillin (50 μg/ml) was added to the medium for the cultivation of *V. parahaemolyticus* or *E. coli* strains, respectively. The bacteria were grown in broth media at 37°C with shaking at 160 rpm, and the growth was measured by the absorbance at 600 nm using a Smart Spec 3000 spectrophotometer (Bio-Rad, Hercules, CA, USA) or by using a standard plate count method [28].

**Table 1:** Bacterial strains and plasmids used in this study.

Strain	Description	Source
*V. parahaemolyticus* strains		
KX-V231	Wild type, serotype O3:K6, KP^ + ^, clinical isolate	[29]
∆VPA1514	KX-V231 ∆VPA1514	This study
∆VPA1514/c1514	KX-V231 ∆VPA1514 strain complemented with pSCB01-VPA1514	This study
*E. coli* strains		
XL1-Blue	*recA1 endA1 gyrA96 thi-1 hsdR17 supE44 relA1 lac* [F’ *proAB lacI*^q^*Z*M15 Tn*10* (Tet^r^)]	GeneMark
SM10λ-*pir*	*thi thr leu tonA lacY supE recA*::RP4–2-Tc::Mu λ *pir*R6K; Km^r^	[50]
AB1157	Wild type, *F*^*-*^ *thr-1 leuB6 proA2 his-4 thi-1 argE2 lacY1 galK2 rspL supE44 ara-14 xyl-15 mtl-1 tsx-33*	[26]
AS391	*sod A, B, and C* mutant, *(sodA:: Mud PR13)25 (sodB-Kan)1-∆2, sodC:: Ω(SpR)*	[26]
AB1157/v	AB1157 strain containing pSCB01	This study
AS391/v	AS391 strain containing pSCB01	This study
AS391/c1514	AS391 strain complemented with pSCB01-VPA1514	This study
Plasmids		
pGEM-T Easy	Cloning vector; Ap^r^	Promega
pDS132	R6K ori *mobRP4 sacB*; Cm^r^	[29]
pDS132-∆VPA1514	pDS132 with VPA1514 deletion	This study
pSCB01	Derived from pBR328 and pDS132; mobRP4; Ap^r^ Cm^r^ Tc^r^	[29]
pSCB01-VPA1514	pSCB01 with VPA1514	This study

### Construction of the deletion mutant

Mutation of VPA1514 gene was generated through overlap extension polymerase chain reaction (PCR) as previously described [27,29]. The bacterial strains and primers used in this study are listed in Tables 1 and 2. Briefly, two DNA fragments were amplified by PCR with primer pairs, VPA1514-M1 and VPA1514-M2, and VPA1514-M3 and VPA1514-M4, from *V. parahaemolyticus* KX-V231 chromosomal DNA. These two amplified fragments were used as templates for a second round of PCR with primers VPA1514-M1 and VPA1514-M4, resulting in the deletion of the VPA1514 gene. The native structural VPA1514 gene was 516 bp in length. An internal fragment of 437 bp was deleted in this mutant gene, leaving 52 and 27 bp in its 5′- and 3′-ends, respectively. This fragment containing the deletion was cloned into the pGEM-T Easy vector, and transformed into *E. coli* XL1-Blue following the manufacturer’s protocol (Promega Co., Madison, WI, USA). The inserted sequence was confirmed by PCR and verified by sequencing. Then, this fragment from the pGEM-T Easy vector was digested by SacI and SphI and cloned into a suicide vector, pDS132, which contained the chloramphenicol resistance gene and the *sacB* gene, conferring sensitivity to sucrose. This plasmid (pDS132-∆VPA1514) was introduced into *E. coli* SM10λ-*pir*, which was mated with *V. parahaemolyticus* strain KX-V231. Thiosulfate-citrate-bile-sucrose (TCBS) agar (Becton, Dickinson Diagnostic Systems) that contained chloramphenicol was used to screen the *V. parahaemolyticus* containing the inserted plasmid. The culture that contained the pDS132-∆VPA1514 plasmid was incubated at 37°C for 3 hours in LB-3% NaCl and then plated onto an LA-3% NaCl with 10% sucrose. The colonies isolated that were unable to grow on LA-3% NaCl that contained chloramphenicol were selected, and the homologous recombination of the deleted fragment was verified by PCR using primers VPA1514-PCR-1-F/VPA1514-PCR-1-R (Table 2).

**Table 2:** Primers used in this study.

Designation	Sequence, 5′ → 3′	Target	Amplicon, bp
VPA1514-M1	TCGTGCTTTCTACCTAGCCC	*V. parahaemolyticus* partial VPA1513 and partial VPA1514	767
VPA1514-M2	ATCACGCCACACACAATGCGGTGTCATCACGAAAGCAGCC		
VPA1514-M3	GGCTGCTTTCGTGATGACACCGCATTGTGTGTGGCGTGAT	*V. parahaemolyticus* partial VPA1514 and partial VPA1515	292
VPA1514-M4	ATGCACTTCGGCATAGTCCG		
VPA1514-C1	GAAGCACCAACACCAGCTAC	*V. parahaemolyticus* partial VPA1513, VPA1514, and partial VPA1515	1,330
VPA1514-C2	CCACATAGAGCTGAATGCGC		
VPA1514-PCR-1-F	GGCATTGAGCCTGTTGTACC	Confirming deletion of VPA1514	1,708
VPA1514-PCR-1-R	CGCCGAAACCATGCAAAGTC		
VPA1514 PCR-2-F	GCTGCTTTCGTGATGACACC	Confirming deletion of VPA1514	473
VPA1514 PCR-2-R	ACGCCACACACAATGCGTG		
VP 16S rRNA-F	TCCCTAGCTGGTCTGAGA	*V. parahaemolyticus* 16S rRNA gene	222
VP 16S rRNA-R	GGTGCTTCTTCTGTCGCT		

### Construction of complementary strain

The entire length of the VPA1514 gene was amplified by PCR with *V. parahaemolyticus* KX-V231 chromosomal DNA as the template using primer pairs VPA1514-C1 and VPA1514-C2; these primers partially overlapped the structural gene sequences of VPA1513 and VPA1515 (Table 2). The amplicon was 1,330 bp in length and consisted of 640 and 174 bp of the upstream and downstream sequences of the structural gene of VPA1514, respectively. The amplicon was then ligated to the pGEMT-easy vector, transformed into *E. coli* XL-1 blue strain and confirmed by PCR and sequencing. Then, the complete VPA1514 gene was cut with SalI and SphI, and ligated to the shuttle vector pSCB01, which had been digested with the same enzymes [27,29]. The plasmid pSCB01-VPA1514, containing the complete sequence of the VPA1514 gene, was propagated in *E. coli* SM10 λ-*pir* and conjugated to the corresponding VPA1514 mutant to generate a complementary strain, which was selected by chloramphenicol resistance (Table 1). The presence of the VPA1514 gene in the strain was confirmed by PCR using primers VPA1514 PCR-2-F/ VPA1514 PCR-2-R (Table 2). Analysis of amino acid sequence alignment was conducted using Vector NTI software version 11.5 (Invitrogen, Carlsbad, CA, USA).

To assay the function of VPA1514 gene in *E. coli*, the plasmid pSCB01-VPA1514 was transformed into the *E. coli* AS391 strain (Table 1) using the calcium chloride method [30]. The complementary *E. coli* AS391/c1514 strain was selected by ampicillin resistance and confirmed by PCR using primers VPA1514-C1 and VPA1514-C2 (Table 2). The control strains *E. coli* AB1157/v and AS391/v (Table 1) were transformed with the cloning vector pSCB01 and selected by ampicillin resistance.

### Inhibition of bacterial growth under different stresses

The inhibition of growth of various strains of *V. parahaemolyticus* by H_2_O_2_ (Santoku Chemical Industries, Tokyo, Japan), menadione (Sigma-Aldrich, Saint Louis, MO, USA), acetic acid (ALPS Chemical Co., Taipei, Taiwan), lactic acid (Nacalai Tesque, Kyoto, Japan) and sodium hypochlorite (J.T. Baker, Mexico City, Mexico) was assayed using broth culture and the disk diffusion method [29]. Cultures of different *V. parahaemolyticus* strains in the mid-exponential phase were spread on a bacterial lawn on LA-3% NaCl agar, to which paper disks (6 mm, Creative Media Products, Taiwan) that had absorbed 10 μl of the specified chemical agents (0.1, 1 or 10 M of H_2_O_2_; 0.01, 0.05 or 0.1 M of menadione; 1 or 10 M of acetic acid; 0.5 or 5 M of lactic acid; 1, 3 or 5% of NaClO) had been placed. The sizes of the inhibition zones were measured following incubation at 37°C for 16 hours. To assay the growth of the *V. parahaemolyticus* or *E. coli* strains in broth culture, bacterial cultures in the exponential phase were resuspended in LB-3% NaCl or LB, respectively, and adjusted to an absorbance of 0.1 at 590 nm, and aliquots (200 μl) were dispensed into the wells of a microplate. Hydrogen peroxide (300 or 350 μM), menadione (30, 50 or 60 μM) or acetic acid (25 or 30 mM for *V. parahaemolyticus*, and 60 mM for *E. coli*) was added. The cultures in the microplate were incubated at 37°C in a static state and bacterial growth was determined by measuring the absorbance at 600 nm using a 96-well microplate reader (BioTek, Winooski, VT, USA).

### Determination of the survival of bacteria under lethal stresses

To evaluate the bactericidal effect of various stresses on *V. parahaemolyticus* strains in LB-3% NaCl, a final concentration of 500 μM H_2_O_2_, 140 μM menadione, 17.5 mM lactic acid or 25 or 30 mM acetic acid (with or without additional 10 μM menadione or 0.2 mM sodium pyruvate) was added to the cultures in the mid-exponential phase which had been adjusted to an absorbance of about 0.1 at 590 nm, and incubated at 37°C. The concentrations of chemical stress agents used herein were determined in preliminary experiments in order to yield lethality in about three hours, while the concentrations of the acetic acid used in this study were similar to those used in food processing [31]. In a control experiment, the survival rates of these strains were determined in LB-3% NaCl which was acidified to pH 4.2 by adding HCl. The survivors were counted at different intervals using the standard plate count method on LA-3% NaCl after incubation at 37°C for 16 hours. The bactericidal effect of 60 mM acetic acid on *E. coli* strains in LB to yield similar lethality to that of *V. parahaemolyticus* in three hours was also determined, and survivors were counted using the LA medium.

The wild-type and ∆VPA1514 mutant strains were cultured in LB-3% NaCl with/without 5 mM acetic acid for 2.5 hours; these cultures were then challenged with 30 mM acetic acid for one hour, and the survivors were counted to determine the effects of adaptation to acetic acid.

### Reverse-transcription Polymerase Chain Reaction

The expression of genes in the *V. parahaemolyticus* strains was determined using reverse-transcription polymerase chain reaction (RT-PCR). Briefly, bacterial strains were cultivated statically in LB-3% NaCl at 37°C, and the cultures in exponential or stationary phase were challenged with 30 mM acetic acid for 1.5 hours. Bacterial cells were harvested by centrifugation and lysed using TRIzol reagent (Invitrogen), and RNA samples were extracted using an RNApure kit (Genesis Biotech Inc., Taipei, Taiwan), following the manufacturer’s instructions. RNA samples were treated with DNase I (TaKaRa Bio Inc., Shiga, Japan) and then reverse transcribed using SuperScript III first-strand synthesis SuperMix (Invitrogen), following the manufacturer’s instructions. PCR was conducted using recombinant Taq DNA polymerase (Ampliqon, Copenhagen, Denmark) with the primers listed in Table 2. 16S ribosomal RNA was used as an internal control, and the primers were described previously [28]. The reactions were heated at 94°C for 5 minutes and then immediately cycled 30 times through a 60-second denaturing step at 94°C, a 60-second annealing step at 55°C (VPA1514) or 58°C (16S rRNA), and a 60-second extension step at 72°C. After the cycling procedure, a final 5-minute elongation step at 72°C was performed. The amplified fragments were resolved by agarose gel electrophoresis. GoalBio 1kb DNA ladder molecular weight marker (Taipei, Taiwan) was used in this assay.

### Statistical analysis

For the bacterial growth and survival experiments, two or three different bacterial cultures were made for *V. parahaemolyticus* strains or *E. coli* strains, respectively, and sampling was conducted in triplicate. The data were analyzed by performing one-way ANOVA with Duncan’s multiple range test at a significance level of α = 0.05, using SPSS for Windows version 11.0 (SPSS Inc., Chicago, IL, USA).

## Results

### Growth of wild-type, ∆VPA1514 mutant and complementary strains of *V. parahaemolyticus*

Based on alignment analysis, the amino acid sequence of VPA1514, which was derived from its nucleotide sequence as determined in *V. parahaemolyticus* KX-V231 in this study, is identical to that of the reference strain RIMD 2210633 (GenBank ID: NP801024) (S1A Fig). In this study, the VPA1514 mutant (∆VPA1514) of *V. parahaemolyticus* KX-V231 and its gene complementary strain (∆VPA1514/c1514) were constructed (Table 1) and verified by polymerase chain reaction (PCR) and sequencing (S1 Fig). The growth of wild-type *V. parahaemolyticus*, its ∆VPA1514 mutant, and the complementary strain in LB-3% NaCl at 37°C was monitored with shaking for 8 hours. These cultures reached the stationary phase at 4 hours with a maximal cell density of about 10^9^ CFU/mL; no significant difference in growth was observed in these strains (S2 Fig).

### Bacterial growth under various stresses

**Fig 1:**
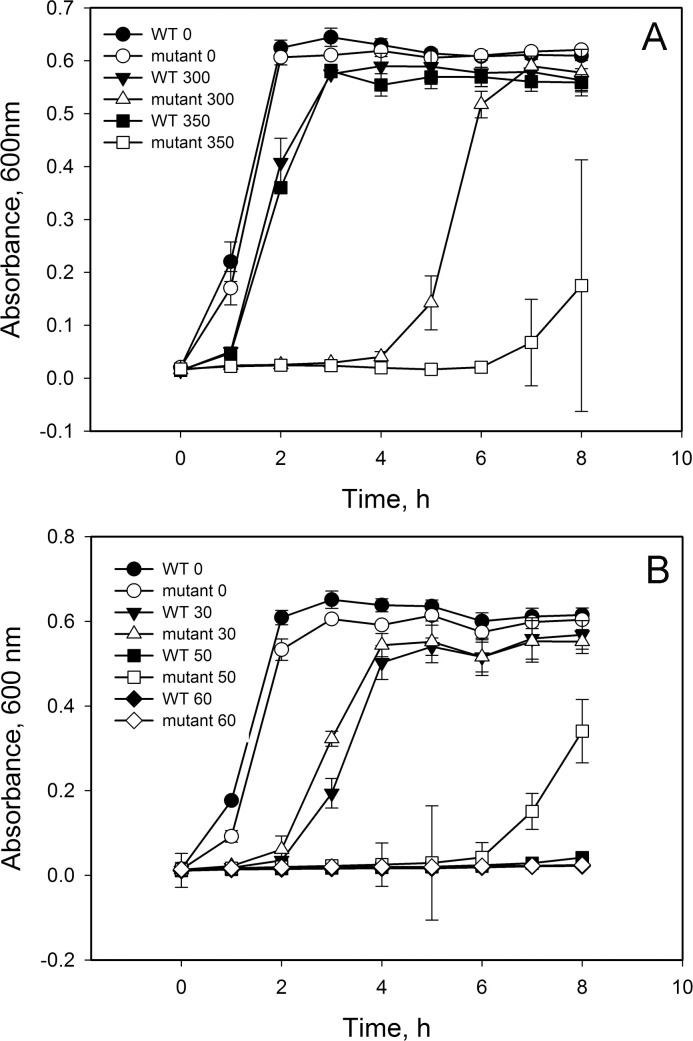
Growth of *V. parahaemolyticus* wild-type KX-V231 and VPA1514 mutant strains under hydrogen peroxide (A) or menadione (B) stress. The growth of *V. parahaemolyticus* wild-type KX-V231 and VPA1514 mutant was determined in LB-3% NaCl at 37°C with or without the addition of H_2_O_2_ or menadione. Panel A: ●, wild-type/no H_2_O_2_; ○, △VPA1514/no H_2_O_2_; ▼, wild-type/300 μM H_2_O_2_; △, △VPA1514/300 μM H_2_O_2_; ■, wild-type/350 μM H_2_O_2_; □, △VPA1514/350 μM H_2_O_2_. Panel B: ●, wild-type/no menadione; ○, △VPA1514/no menadione; ▼, wild-type/30 μM menadione; △, △VPA1514/30 μM menadione; ■, wild-type/50 μM menadione; □, △VPA1514/50 μM menadione; ♦, wild-type/60 μM menadione; ⋄, △VPA1514/60 μM menadione. Data shown are the mean ± SE from three independent experiments.

The influence of different stresses on the growth of these strains was determined in broth and agar medium. H_2_O_2_, acetic acid, lactic acid and sodium hypochlorite are commonly used as disinfectants/sanitizers, acidulants or preservatives in the food industry, while menadione is a superoxide generator used to assay the function of SODs [32]. In this study, the effect of 300 and 350 μM H_2_O_2_ or 30, 50, and 60 μM menadione on the growth of the strains of interest in static broth culture was examined. No significant difference in growth was observed in the wild-type and mutant strains when no chemical stress agent was applied (Fig 1). The presence of H_2_O_2_ slowed the growth of these strains, whereas the growth of wild-type strain was delayed by about one hour by 300 or 350 μM H_2_O_2_ with no significant difference between these two concentrations. The ∆VPA1514 mutant strain exhibited a longer lag period than the wild-type strain to reach stationary phase, and it was more sensitive in response to 350 μM than to 300 μM H_2_O_2_ (Fig 1A). The growth of wild-type and mutant strains in broth medium with 30 μM or 60 μM menadione did not show significant difference; however, the ∆VPA1514 mutant resumed bacterial growth sooner than the wild-type strain when challenged with 50 μM menadione (Fig 1B).

As determined by the disk diffusion method, the growth of the wild-type and ∆VPA1514 mutant strains on agar medium was inhibited by H_2_O_2_, menadione, acetic acid, lactic acid and sodium hypochlorite (NaClO) at the specified concentrations. However, the inhibition of growth of the ∆VPA1514 mutant did not significantly differ from that of the wild-type strain (S3 Fig). Therefore, these assays did not demonstrate a protective role for VPA1514 against these stresses at these concentrations.

### Survival of wild-type, ∆VPA1514 mutant, and complementary strains under lethal stresses

**Fig 2:**
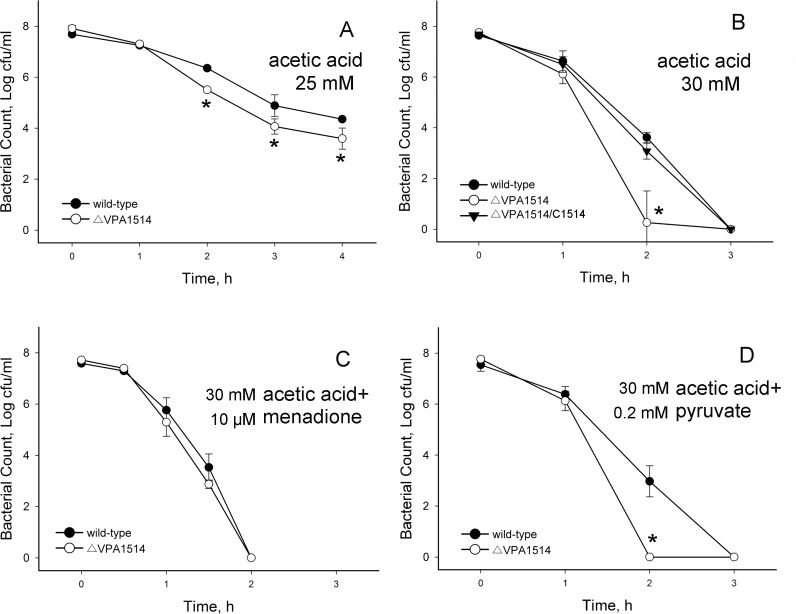
Survival of different *V. parahaemolyticus* strains under lethal chemical stresses. *V. parahaemolyticus* cultures in the exponential phase were challenged with 25 mM acetic acid (A), 30 mM acetic acid (pH 4.73) (B), 30 mM acetic acid plus 10 μM menadione (C), or 30 mM acetic acid plus 0.2 mM sodium pyruvate (D). Cell viability was determined using a standard plate count method at intervals. ●, wild-type KX-V231; ○, VPA1514 mutant; ▼, VPA1514 mutant with complementary gene (strain ∆VPA1514/c1514). Data shown are the mean ± SE from three independent experiments. Unpaired t-tests were used to calculate P values. (*, p < 0.05).

Next, rates of survival of *V. parahaemolyticus* strains under lethal chemical stresses in broth medium were determined. Under challenge by 25 mM acetic acid, the ∆VPA1514 mutant strain showed significantly lower survival rates than those of the wild-type strain in two to four hours (Fig 2A). Thirty millimolar acetic acid (pH 4.73) (Fig 2B) and 17.5 mM lactic acid (pH 4.79) (S4C Fig) in the culture media provided similar acidities and similar bactericidal activities in the wild-type strain. When the concentration of acetic acid was raised to 30 mM, survival in these strains was undetectable at three hours, but the survival of the ∆VPA1514 mutant strain when challenged for two hours was approximately 10^3^ CFU/ml lower than that of the wild-type strain. The presence of the complementary VPA1514 gene significantly increased the survival rate of this mutant strain at two hours (Fig 2B).

Menadione at 10 μM did not inhibit the growth of *V. parahaemolyticus* strains in this study, but the addition of 10 μM menadione potentiated the lethal activity of 30 mM acetic acid against both the wild-type and the ∆VPA1514 mutant strains, which had no survival after two hours (Fig 2C). The results indicate that VPA1514 may not have an effective role in this experiment. The addition of 0.2 mM sodium pyruvate, a peroxide scavenger [33], did not change the lethal activity of the acetic acid (Fig 2D), and this result is similar to those shown in Fig 2B. Since the function of VPA1514 was limited in the experiments shown in Fig 2C and 2D, the complementary strain was not examined in these two experiments.

**Fig 3:**
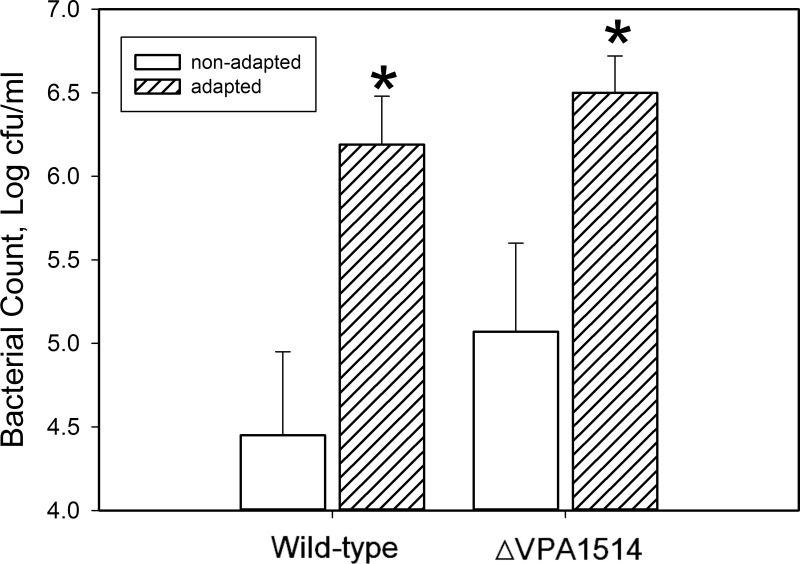
Effect of acetic acid adaptation on the survival of *V. parahaemolyticus* strains under lethal acetic acid challenge. *V. parahaemolyticus* cultures were adapted to 5 mM acetic acid for 2.5 hours and challenged with 30 mM acetic acid for one hour. Cell viability was determined and is presented as means with standard deviations. Open bar, non-adapted cultures; shaded bar, adapted cultures. Adaptation significantly enhanced survival in both wild-type and ∆VPA1514 mutant strains, as indicated by an asterisk (p < 0.05). No significant difference was observed between non-adapted strains or between adapted strains.

The wild-type and ∆VPA1514 mutant strains were adapted to a sublethal concentration of acetic acid (5 mM) for 2.5 hours, during which no decrease in bacterial counts was observed. After subsequent challenge with a lethal concentration of acetic acid (30 mM), the survival rates of both strains increased significantly. No significant differences were observed between the wild-type and ∆VPA1514 mutant strains in either the adapted or non-adapted state. This result indicates that the VPA1514 gene is not responsive to this adaptation process (Fig 3).

Under challenge by 500 μM H_2_O_2_ or 140 μM menadione for four hours, the number of viable cells of wild-type and ∆VPA1514 was about 10^2^ CFU/ml in the H_2_O_2_ group (S4A Fig) or 10^4^ CFU/ml in the menadione group (S4B Fig). The lactic acid (17.5 mM) treatment killed all the bacteria in three hours, and no significant difference was found in the survival rates of wild-type and ∆VPA1514 mutant strains (S4C Fig). No significant difference in survival was observed among the wild-type, ∆VPA1514 mutant, and the gene-complementary strains by lowering the acidity of the broth to pH 4.2 by adding inorganic acid HCl (S4D Fig).

### Expression of the VPA1514 gene in *V. parahaemolyticus* strains under challenge by acetic acid

**Fig 4:**
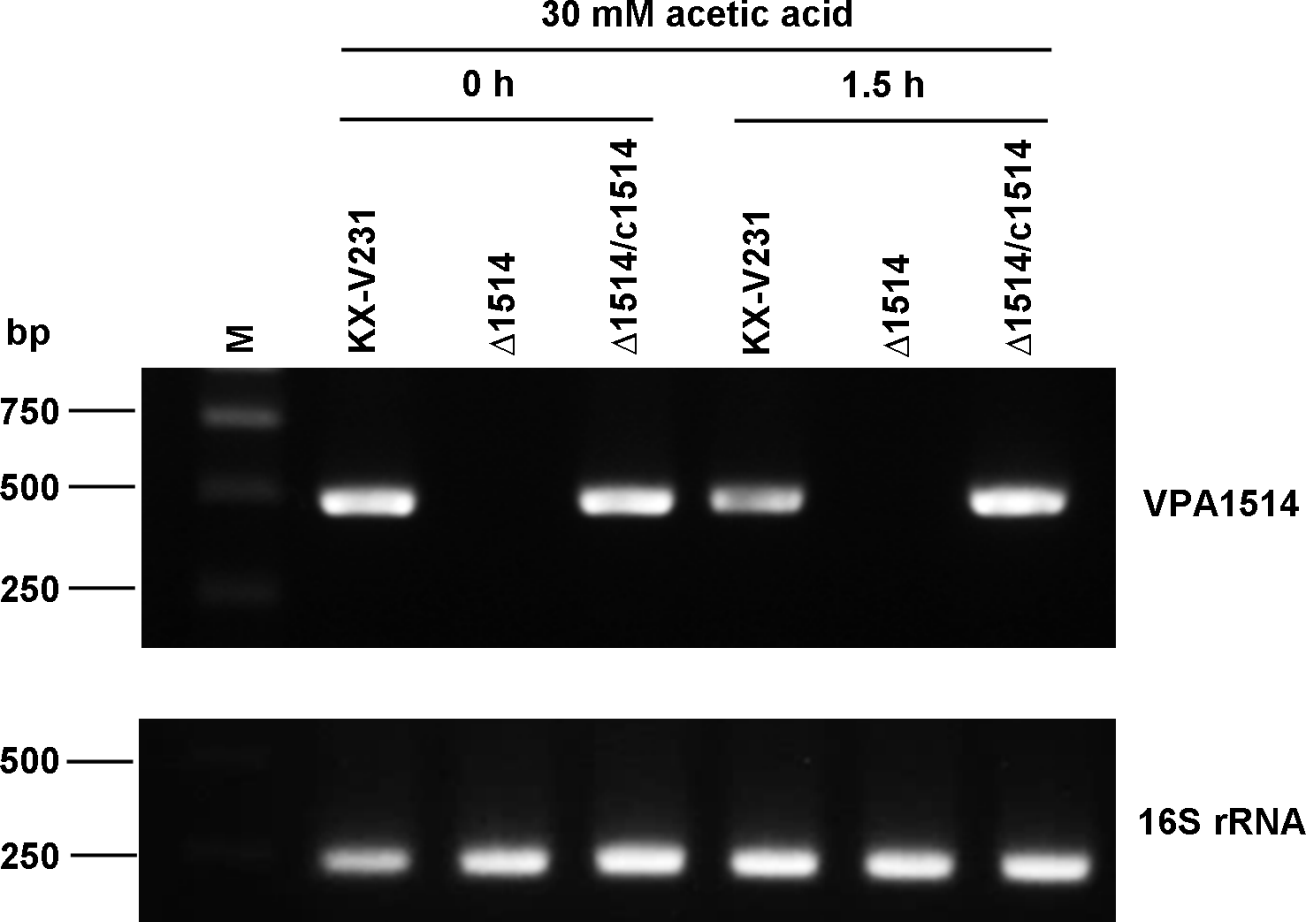
VPA1514 gene expression in wild-type, ∆VPA1514 mutant, and complementary strains of *V. parahaemolyticus* under challenge by acetic acid. Bacterial cultures in the exponential phase were challenged with 30 mM acetic acid for 1.5 hours and VPA1514 gene expression was determined by RT–PCR. 16S rRNA was used as control; M, molecular size marker.

The expression of the VPA1514 gene in the wild-type, ∆VPA1514 mutant, and complementary strains of *V. parahaemolyticus* during a challenge by acetic acid was determined by RT-PCR. In the wild-type and complementary strains, the expression of the VPA1514 gene was not markedly enhanced by treatment with 30 mM acetic acid for 1.5 hours (Fig 4). Moreover, the VPA1514 gene was not detectable in the ∆VPA1514 strain (Fig 4). Expression of VPA1514 in the wild-type strain maintained a similar level in the exponential phase and stationary phase, and was not stimulated by acetic acid stress (S5 Fig).

### Protection of VPA1514 complementary gene against acetic acid in SODs mutant of *E. coli*

To examine the contribution of VPA1514 to the resistance of bacterial cells to acetic acid, the heterologous gene complementary *E. coli* AS391/c1514 strain was constructed in AS391. VPA1514 was then expressed in an *E. coli* strain that lacked endogenous FeSOD, MnSOD and CuZnSOD genes. Wild-type *E. coli* and AS391 strains were also transformed with the cloning vector that is used in this study. The growth of these *E. coli* strains was examined in LB at 37°C in shaking or static cultures for 8 hours. No significant difference was observed between the growth of the SODs mutant and that of the VPA1514 complementary strains under these culture conditions (S6 Fig).

In a preliminary study, the growth of these *E. coli* strains was completely inhibited by 12.5 mM acetic acid in a broth culture. Under a challenge by 6.25 mM acetic acid, the growth of the SODs mutant strain of *E. coli* was slowed relative to that of the wild-type strain, and such slowed growth was not subsequently ameliorated by the presence of the complementary VPA1514 gene (S7 Fig).

**Fig 5:**
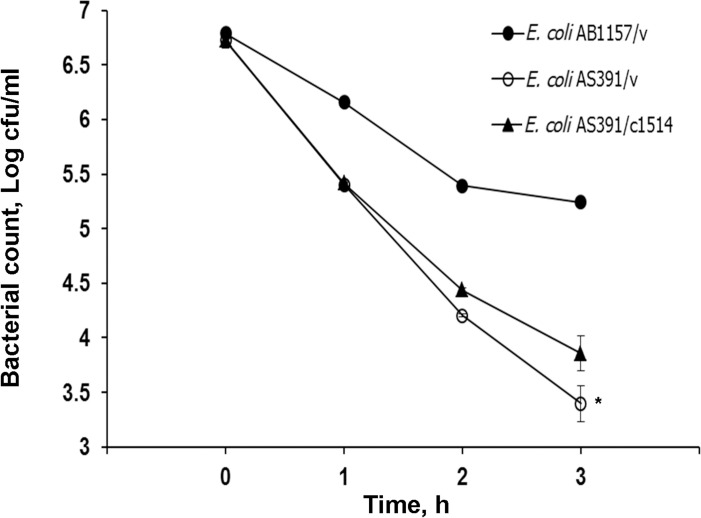
Effect of VPA1514 complementation on the survival of *E. coli* SODs mutant strain challenged with acetic acid. *E. coli* cultures in LB were challenged with 60 mM acetic acid at 37°C, and cell viability was determined. The means of cell survival at each time point were compared using a t-test. The asterisk indicates significant difference (p < 0.05) between the SODs mutant and the VPA1514 complementary strain. ●, *E. coli* AB1157/v (wild-type strain with cloning vector); ○, *E. coli* AS391/v (*sod A, B, and C* mutant with cloning vector); ▲, *E. coli* AS391/c1514 (SODs mutant with complementary VPA1514 complementation).

When *E. coli* strains were challenged by a lethal concentration of acetic acid (60 mM) for 3 hours, the survival of the SODs mutant strain was lower than that of the wild-type strain (Fig 5). Furthermore, the presence of VPA1514 gene significantly increased the survival rate of the *E. coli* mutant strain following acetic acid treatment (Fig 5). These results demonstrated that the VPA1514 gene of *V. parahaemolyticus* could not restore the protective function of the SODs in this *E. coli* mutant strain under growth inhibitory stress, but it contributes to the resistance of bacteria against lethal treatment with acetic acid.

## Discussion

Periplasmic CuZnSOD is responsible for the antioxidative function against the oxidative burst of phagocytes in pathogenic bacteria [26,34,35] and is associated with the virulence of *Salmonella* [36] and *E. coli* [37]. It has been demonstrated that the periplasmic CuZnSOD defends bacteria against exogenous hydrogen peroxide [38]. CuZnSOD also defends against superoxides [39,40] by blocking the influx of external superoxides into the cytosol of Gram-negative bacteria [41]. The periplasmic CuZnSOD may also participate in the tolerance of bacteria against different environmental stresses. A yeast CuZnSOD protects *E. coli* against other environmental stresses, such as heat shock and superoxide-generating agents, such as paraquat and menadione [39].

The CuZnSOD of *V. parahaemolyticus* is composed of 171 amino acids and has a calculated molecular weight of 18.81 kDa. Alignment of the CuZnSOD amino acid sequence from *V. parahaemolyticus* with some homologous CuZnSODs reveals conserved histidine residues for Cu binding and the histidine and aspartic acid residues for Zn binding (S8A Fig) [42].

The CuZnSODs of the *Vibrio* species have not been well characterized and may have undescribed features (S8 Fig). Deletion mutants of FeSOD (VP2118), MnSOD (VP2860) and CuZnSOD (VPA1514) were constructed and it was demonstrated that the VP2118 is the chief SOD enzyme of *V. parahaemolyticus* against peroxide and superoxide, while VPA1514 is not significant against these oxidative stresses [20]. In addition, the comparable effects of menadione on the growth and survival of the △VPA1514 mutant and wild-type strain suggest that VPA1514 is not a key enzyme for superoxide detoxification in *V. parahaemolyticus*. Le *et al*. (2012) suggested that VPA1514 in *V. parahaemolyticus* may have additional roles in the interaction with host cells. This study demonstrated that VPA1514 is capable of treating exogenous H_2_O_2_ and may be helpful for the survival of this pathogen against the oxidative burst of the host [26,43]. Nevertheless, the function of VPA1514 in virulence may not be as critical as that of the CuZnSOD in closely related species, such as *V. vulnificus*, and it appears to be less significantly associated with virulence than the other two cytosolic SODs [44].

This study demonstrated the protective function of VPA1514 of *V. parahaemolyticus* against H_2_O_2_ in broth medium. These results suggest that VPA1514 is the first barrier against exogenous H_2_O_2_, and other compensatory enzymes may be induced during the lag period of the △VPA1514 mutant under H_2_O_2_ stress. The H_2_O_2_ protective function of VPA1514 was not observed in the disk diffusion assay, which may be attributed to the difference in the agar or broth medium and length of culture time.

This study demonstrates that CuZnSOD (VPA1514) contributes to the survival of *V. parahaemolyticus* under lethal acetic acid stress. Acetic acid, a common food acidulant and preservative, is toxic to bacterial cells due to its dissociation inside microbial cells, causing a decrease in intracellular acidity and metabolic disturbance [45]. The concentration of acetic acid used in this study is comparable to the levels typically applied in food processing. Thus, CuZnSOD may influence the effectiveness of acetic acid as a food preservative against *V. parahaemolyticus*.

The role of VPA1514 against lethal acetic acid treatment was also verified in the SOD mutant strain of *E. coli* AS391. Expression of different *V. parahaemolyticus* genes using their native promoters in *E. coli* has been demonstrated in other studies [46,47]. Overexpression of VPA1514 in *V. parahaemolyticus* and *E. coli* will further characterize the function of this enzyme.

This study did not find a protective function for VPA1514 against lactic acid. Since the concentrations of acetic acid and lactic acid that are used in this investigation lowered the acidity of the culture broth to similar levels (pH 4.73–4.79) and caused similar lethality in the wild-type strain, the different results of these two organic acids in the VPA1514 mutant strain may not simply be attributable to the low acidities rendered by these organic acids. In fact, bacteria have different metabolic responses to acetic acid and lactic acid [48], whereas the acetic acid-induced tolerance response (ATR) cross-protects *Salmonella* species against osmotic stress, the lactic acid-induced ATR fails in such cross-protection [10].

Although this study addresses the role of a CuZnSOD in the resistance of bacteria to acetic acid stress, the regulation of CuZnSOD or other periplasmic SODs by organic acids requires clarification. Reports concerning the expression of CuZnSOD in *E. coli* are inconsistent. The CuZnSOD gene is positively regulated by the RpoS system in *E. coli* and is strongly induced in the stationary phase [49]. In the present study, the CuZnSOD (VPA1514) gene of *V. parahaemolyticus* was expressed in the exponential and stationary phases and was not markedly dependent on the acetic acid treatment. The main difference between these results may be because of the culturing time to obtaining the stationary phase cultures. In the present study, the bacterial strains were cultured for 4 hours with an OD of about 0.6; these cultures may be in an early stationary phase as compared to those cultures with an OD of 2.4 [49]. As also demonstrated in other studies, the production of CuZnSOD in *Brucella melitensis* [17] and *E. coli* [18] is also not associated with acid stress.

In summary, this study reports on the involvement of CuZnSOD (VPA1514) in the resistance of *V. parahaemolyticus* against H_2_O_2_ and lethal concentrations of acetic acid (30 mM). These results indicate that the CuZnSOD may be important in determining the resistance of *V. parahaemolyticus* to domestic vinegar during food processing.

## Supporting information

S1 Fig Confirmation of VPA1514 in the wild-type, ∆VPA1514 mutant, and complementary strains of *V. parahaemolyticus.* Panel A: Alignment of amino acid sequences of VPA1514 genes in the wild-type strain (KX-V231) and reference strain (RIMD 2210633) of *V. parahaemolyticus* (GenBank ID: NP801024). Panel B: Deletion of VPA1514 gene verified by PCR in the wild-type and ∆VPA1514 mutant strains using the primers VPA1514 PCR-1-F/VPA1514 PCR-1-R. Amplicons of 1,708 bp or 1,231 bp were detected in the wild-type and mutant strains, respectively. Panel C: Presence of VPA1514 gene verified by PCR in the wild-type (KX-V231), ∆VPA1514 mutant and complementary strains (△VPA1514/c1514) of *V. parahaemolyticus*, using the primers VPA1514 PCR-2-F/ VPA1514 PCR-2-R, and only amplicon of 473 bp was detected in the wild-type and complementary strains. M, molecular size marker.
(TIF)

S2 Fig Growth of wild-type, ∆VPA1514 mutant, and complementary strains of *V. parahaemolyticus* in broth medium. Bacterial strains were cultured in LB-3% NaCl at 37°C and shaken at 160 rpm. Bacterial growth was determined by a standard plate count method using LA-3%NaCl plates. ●, wild-type strain; ○, VPA1514 mutant; ▲, VPA1514 mutant with complementary VPA1514 gene. Data shown are the mean ± SE from three independent experiments.
(TIF)

S3 Fig Inhibition of growth of wild-type KX-V231 (solid bars) and VPA1514 mutant strains (open bars) of *V. parahaemolyticus* by different chemical stresses. Paper disks containing 10 μl of the indicated concentrations of H_2_O_2_, menadione, acetic acid, lactic acid or sodium hypochlorite (NaClO) were placed on bacterial lawns on LA-3%NaCl plates. The diameters of the inhibition zones were measured after 16 hours of incubation at 37°C. Data shown are the mean ± SE from three independent experiments.
(TIF)

S4 Fig Survival of wild-type and VPA1514 mutant strains of *V. parahaemolyticus* under lethal chemical stresses of H_2_O_2_, menadione, lactic acid and hydrochloric acid. *V. parahaemolyticus* cultures in the exponential phase were challenged with 500 μM H_2_O_2_ (A), 140 μM menadione (B), 17.5 mM lactic acid (pH 4.79) (C) or in broth acidified to pH 4.2 using hydrochloric acid (D). Survivors were counted using a standard plate count method at intervals. ●, wild-type KX-V231; ○, VPA1514 mutant (strain ∆VPA1514); ▼, VPA1514 mutant with complementary gene (strain ∆VPA1514/c1514). Data shown are the mean ± SE from three independent experiments.
(TIF)

S5 Fig Expression of the VPA1514 gene of the wild-type strain challenged with 30 mM acetic acid was determined in the exponential and stationary phases. Bacterial cultures were cultivated statically in LB-3% NaCl at 37°C for 2 or 4 hours to reach the exponential or stationary phase, respectively, challenged by 30 mM acetic acid for 1.5 hours, and expression of VPA1514 gene was determined by RT–PCR. 16S rRNA was used as a control.
(TIF)

S6 Fig Growth of wild-type, SODs mutant, and VPA1514 complementary strains of *E. coli.* Bacterial strains were cultured in LB broth at 37°C under shaking (A and B) or static conditions (C and D). Bacterial growth was determined by measuring the absorbance of cultures at 600 nm (A and C), and survivors were counted using a standard plate count method (B and D). ●, *E. coli* AB1157/v, wild-type strain containing cloning vector pSCB01; ○, *E. coli* AS391/v, *sodA*, *sodB* and *sodC* mutant containing cloning vector pSCB01; ▲, *E. coli* AS391/c1514, *sod* genes mutant containing complementary VPA1514 gene. Data shown are the mean ± SE from three independent experiments.
(TIF)

S7 Fig Influence of VPA1514 complementary gene on growth *of E. coli* SODs mutant strain. *E. coli* cultures in LB were challenged with 6.25 mM acetic acid at 37°C, and bacterial growth was determined by measuring absorbance at 600 nm. ●, *E. coli* AB1157/v, wild-type strain containing cloning vector pSCB01; ○, *E. coli* AS391/v, *sodA*, *sodB* and *sodC* mutant containing cloning vector pSCB01; ▲, *E. coli* AS391/c1514, the *sod* genes mutant containing complementary VPA1514 gene. Data shown are the mean ± SE from three independent experiments.
(TIF)

S8 Fig Amino acid sequence alignment of CuZnSOD (VPA1514) of *V. parahaemolyticus* and other homologous CuZnSODs (A) and dendrogram analysis of these proteins (B). The amino acid sequences of CuZnSODs of *V. parahaemolyticus* (NP_801024), *V. alginolyticus* (WP_005383991), *V. cholerae* (WP_069731346), *V. vulnificus* (WP_011151698), *E. coli* SodC1 (WP_106898648), *E. coli* SodC2 (WP_000823671), *Salmonella enterica* SodC1 (WP_079785378) and *S. enterica* SodC2 (WP_000826825) were analyzed using Clustal Omega Program (https://www.uniprot.org/). Solid arrows indicate the histidine residues for Cu binding, and open arrows indicate the histidine or aspartic acid residues for Zn binding.
(TIF)

S1 File Raw images. (PDF)
