# Supplementary figures and images for: Superoxide dismutase VPA1514 in Vibrio parahaemolyticus protects against environmental stresses
Source: PLoS One. 2025 Aug 14;20(8):e0329351. doi: 10.1371/journal.pone.0329351 (PMC12352638; doi:10.1371/journal.pone.0329351)

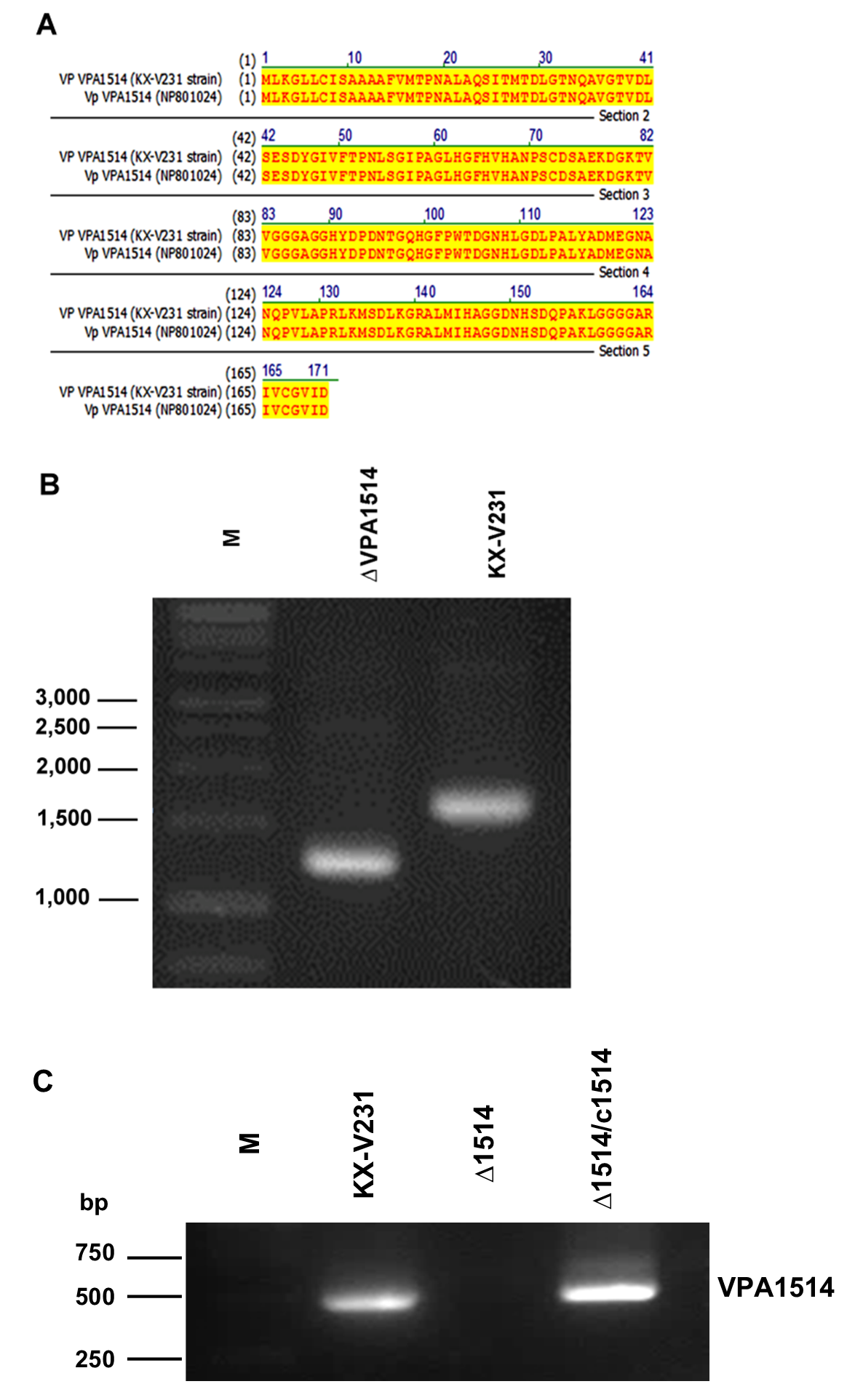

Supplement: S1 Fig — Panel A: Alignment of amino acid sequences of VPA1514 genes in the wild-type strain (KX-V231) and reference strain (RIMD 2210633) of V. parahaemolyticus (GenBank ID: NP801024). Panel B: Deletion of VPA1514 gene verified by PCR in the wild-type and ∆VPA1514 mutant strains using the primers VPA1514 PCR-1-F/VPA1514 PCR-1-R. Amplicons of 1,708 bp or 1,231 bp were detected in the wild-type and mutant strains, respectively. Panel C: Presence of VPA1514 gene verified by PCR in the wild-type (KX-V231), ∆VPA1514 mutant and complementary strains (△VPA1514/c1514) of V. parahaemolyticus, using the primers VPA1514 PCR-2-F/ VPA1514 PCR-2-R, and only amplicon of 473 bp was detected in the wild-type and complementary strains. M, molecular size marker. (TIF) [file pone.0329351.s001.tif]

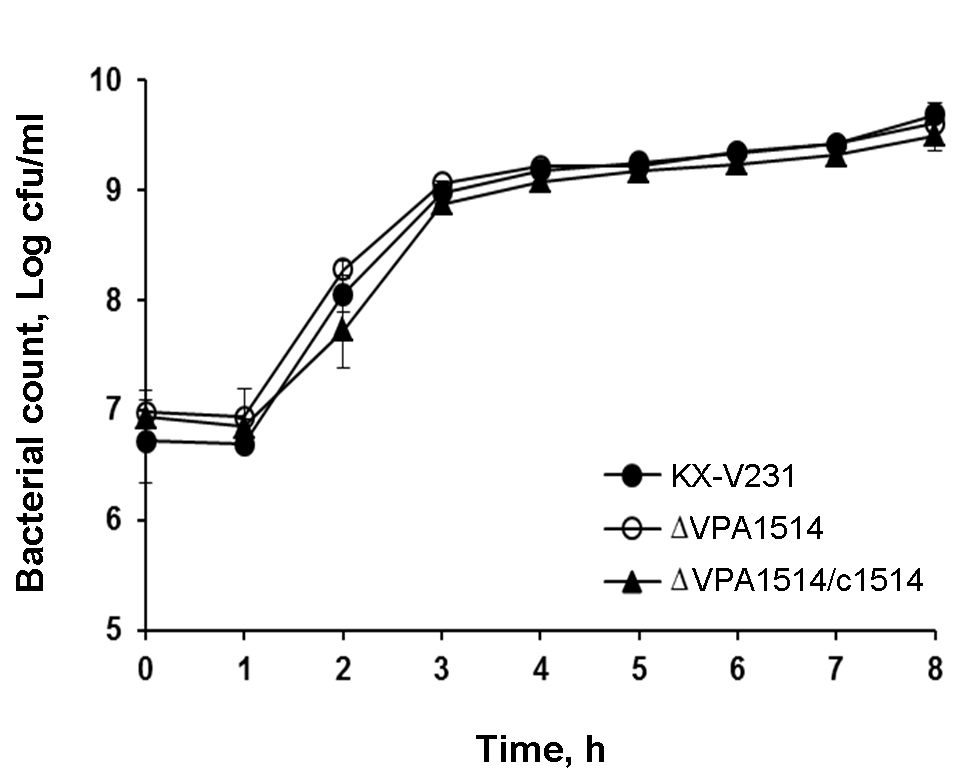

Supplement: S2 Fig — Bacterial strains were cultured in LB-3% NaCl at 37°C and shaken at 160 rpm. Bacterial growth was determined by a standard plate count method using LA-3%NaCl plates. ●, wild-type strain; ○, VPA1514 mutant; ▲, VPA1514 mutant with complementary VPA1514 gene. Data shown are the mean ± SE from three independent experiments. (TIF) [file pone.0329351.s002.tif]

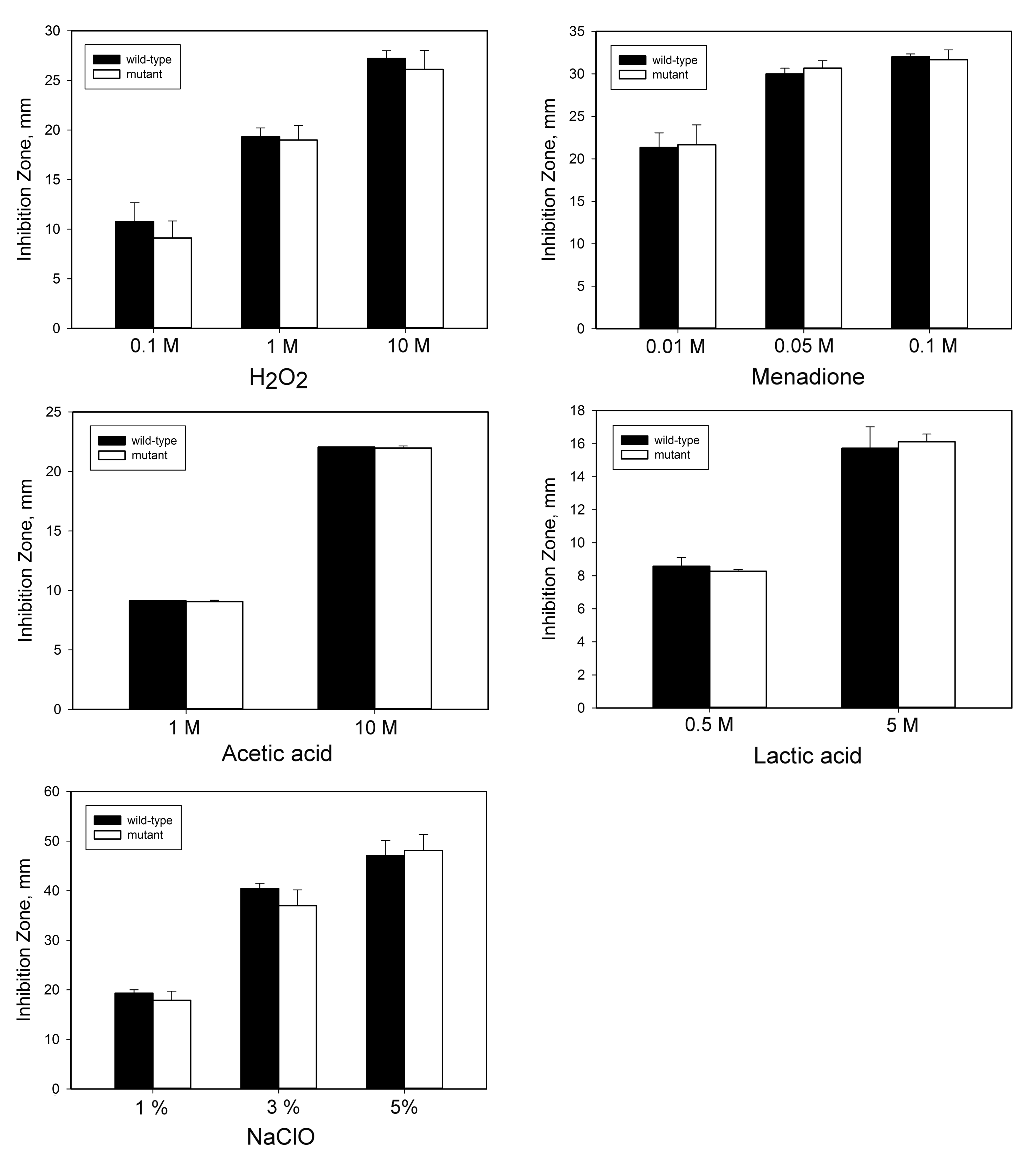

Supplement: S3 Fig — Paper disks containing 10 μl of the indicated concentrations of H2O2, menadione, acetic acid, lactic acid or sodium hypochlorite (NaClO) were placed on bacterial lawns on LA-3%NaCl plates. The diameters of the inhibition zones were measured after 16 hours of incubation at 37°C. Data shown are the mean ± SE from three independent experiments. (TIF) [file pone.0329351.s003.tif]

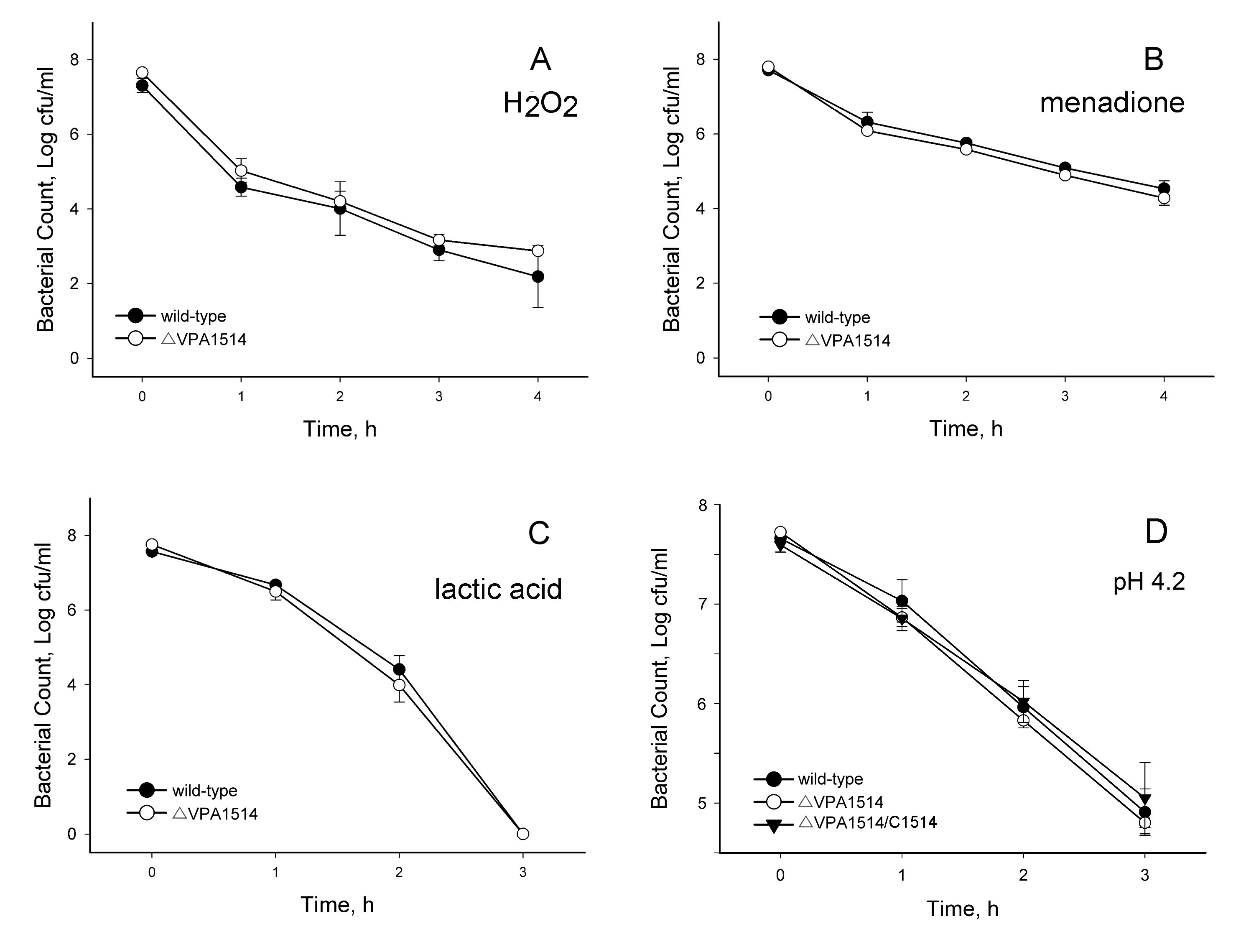

Supplement: S4 Fig — V. parahaemolyticus cultures in the exponential phase were challenged with 500 μM H2O2 (A), 140 μM menadione (B), 17.5 mM lactic acid (pH 4.79) (C) or in broth acidified to pH 4.2 using hydrochloric acid (D). Survivors were counted using a standard plate count method at intervals. ●, wild-type KX-V231; ○, VPA1514 mutant (strain ∆VPA1514); ▼, VPA1514 mutant with complementary gene (strain ∆VPA1514/c1514). Data shown are the mean ± SE from three independent experiments. (TIF) [file pone.0329351.s004.tif]

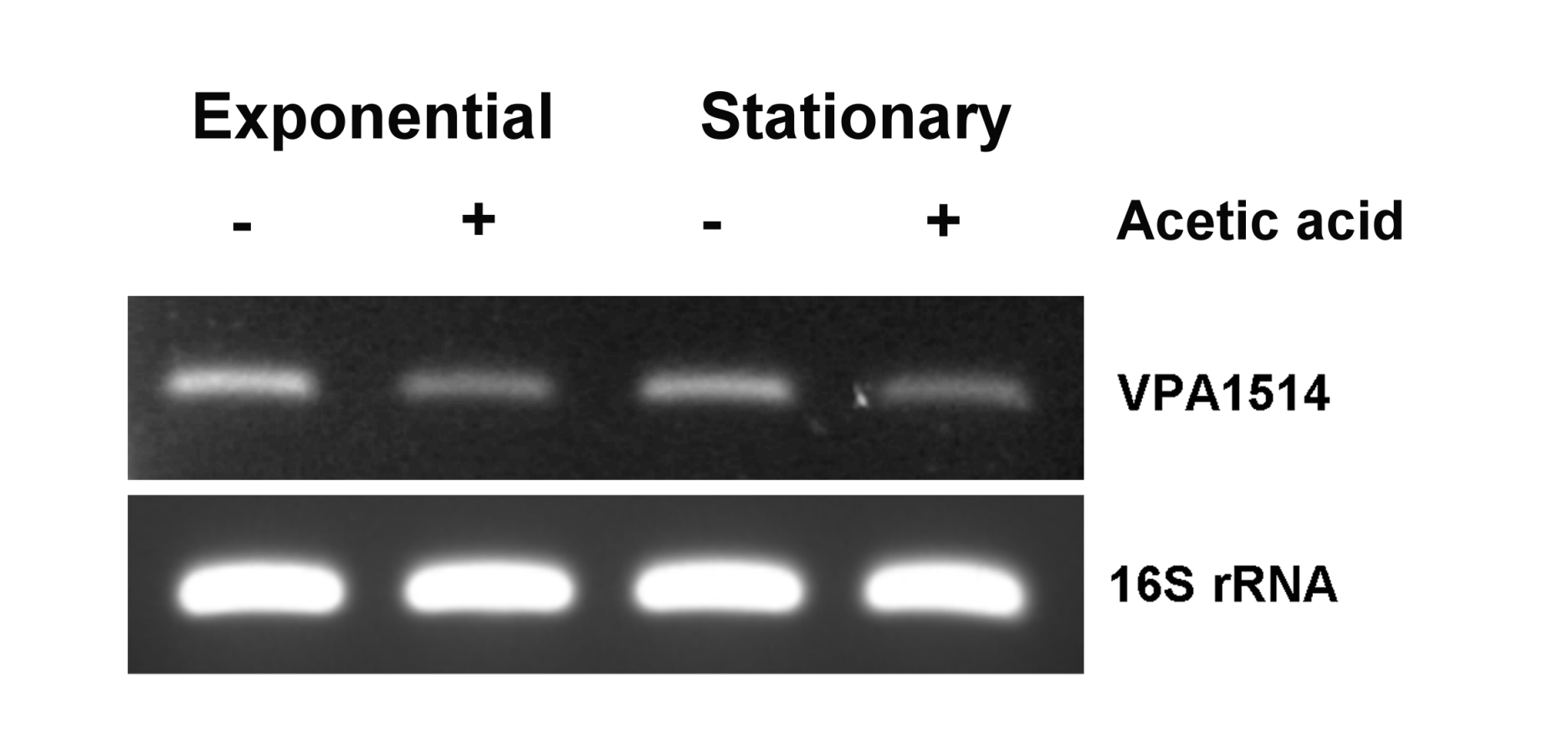

Supplement: S5 Fig — Bacterial cultures were cultivated statically in LB-3% NaCl at 37°C for 2 or 4 hours to reach the exponential or stationary phase, respectively, challenged by 30 mM acetic acid for 1.5 hours, and expression of VPA1514 gene was determined by RT–PCR. 16S rRNA was used as a control. (TIF) [file pone.0329351.s005.tif]

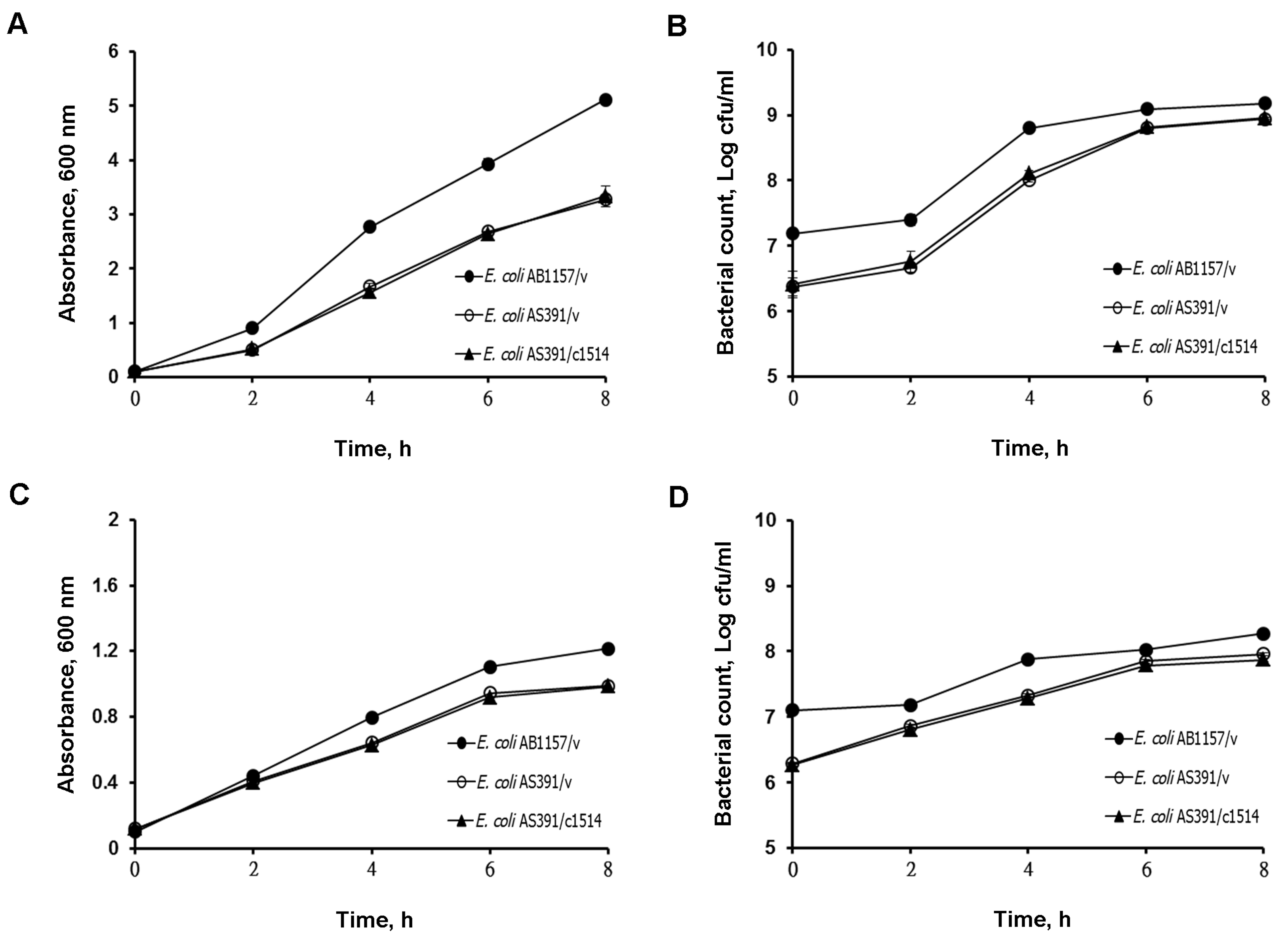

Supplement: S6 Fig — Bacterial strains were cultured in LB broth at 37°C under shaking (A and B) or static conditions (C and D). Bacterial growth was determined by measuring the absorbance of cultures at 600 nm (A and C), and survivors were counted using a standard plate count method (B and D). ●, E. coli AB1157/v, wild-type strain containing cloning vector pSCB01; ○, E. coli AS391/v, sodA, sodB and sodC mutant containing cloning vector pSCB01; ▲, E. coli AS391/c1514, sod genes mutant containing complementary VPA1514 gene. Data shown are the mean ± SE from three independent experiments. (TIF) [file pone.0329351.s006.tif]

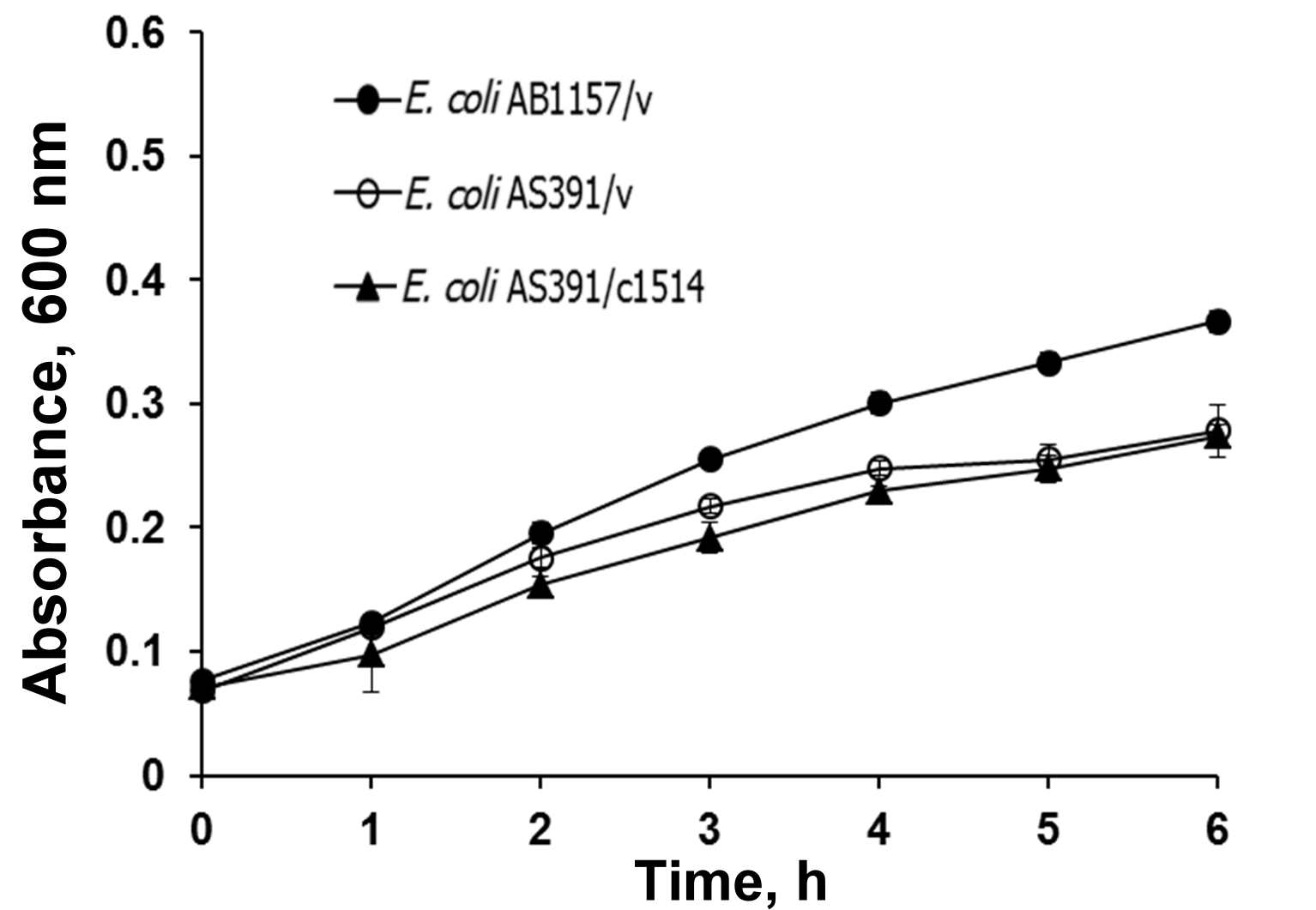

Supplement: S7 Fig — E. coli cultures in LB were challenged with 6.25 mM acetic acid at 37°C, and bacterial growth was determined by measuring absorbance at 600 nm. ●, E. coli AB1157/v, wild-type strain containing cloning vector pSCB01; ○, E. coli AS391/v, sodA, sodB and sodC mutant containing cloning vector pSCB01; ▲, E. coli AS391/c1514, the sod genes mutant containing complementary VPA1514 gene. Data shown are the mean ± SE from three independent experiments. (TIF) [file pone.0329351.s007.tif]

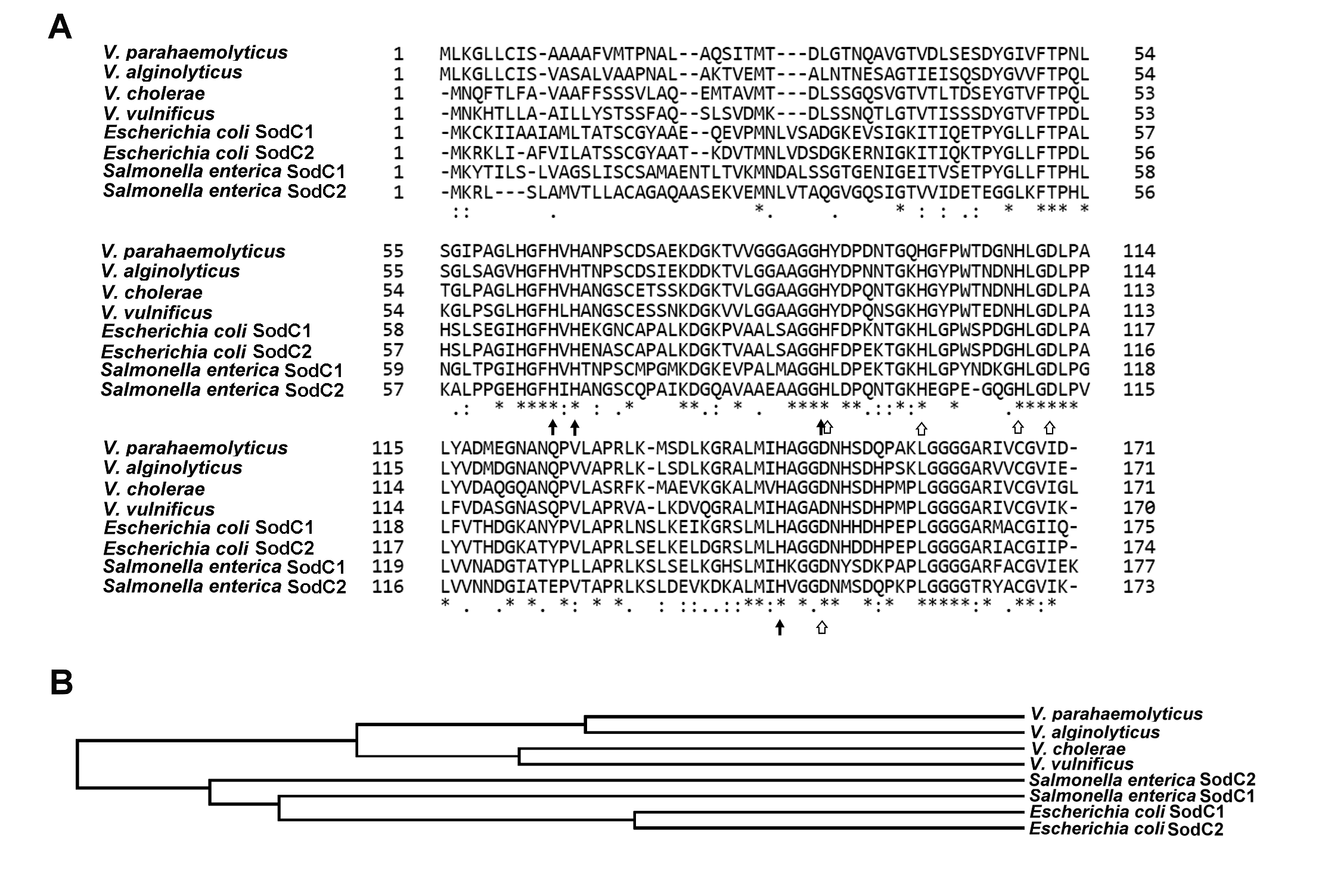

Supplement: S8 Fig — The amino acid sequences of CuZnSODs of V. parahaemolyticus (NP_801024), V. alginolyticus (WP_005383991), V. cholerae (WP_069731346), V. vulnificus (WP_011151698), E. coli SodC1 (WP_106898648), E. coli SodC2 (WP_000823671), Salmonella enterica SodC1 (WP_079785378) and S. enterica SodC2 (WP_000826825) were analyzed using Clustal Omega Program (https://www.uniprot.org/). Solid arrows indicate the histidine residues for Cu binding, and open arrows indicate the histidine or aspartic acid residues for Zn binding. (TIF) [file pone.0329351.s008.tif]
