# Supplementary material for: Superoxide dismutase VPA1514 in Vibrio parahaemolyticus protects against environmental stresses
Source: PLoS One. 2025 Aug 14;20(8):e0329351. doi: 10.1371/journal.pone.0329351 (PMC12352638; doi:10.1371/journal.pone.0329351)
Supplement: S1 File — (PDF) [file pone.0329351.s009.pdf]

## Information on Raw Gel Images

(A)

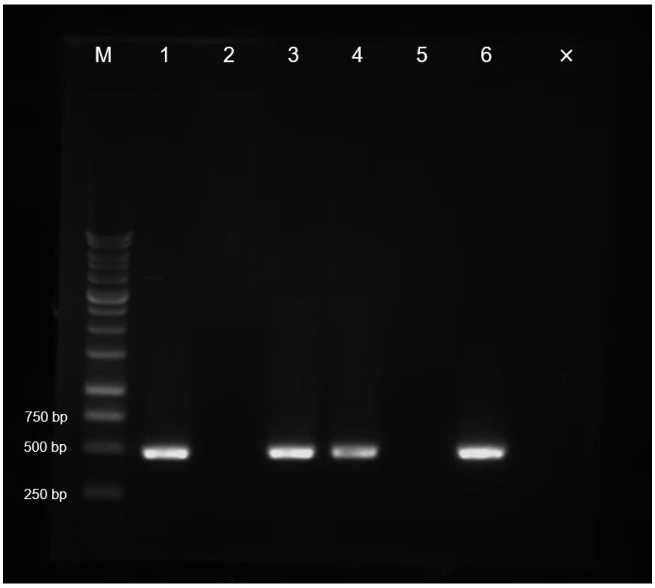


(B)

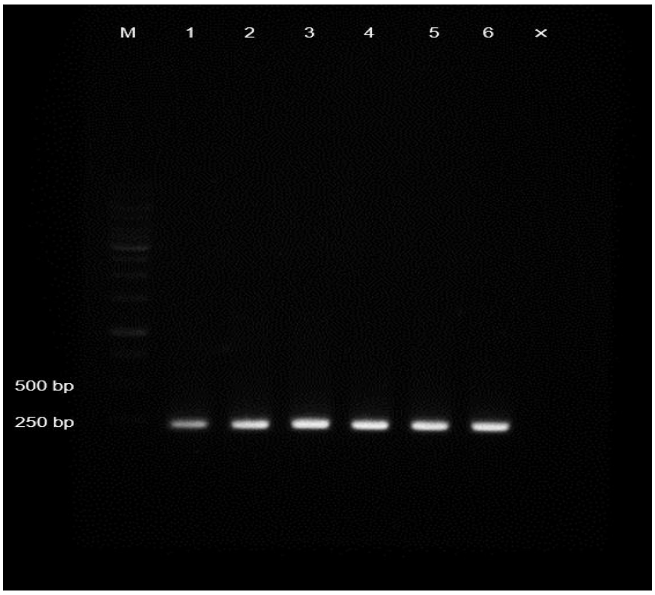


**Fig 4 original image.** RT–PCR analysis of VPA1514 gene expression in the wild-type, ΔVPA1514 mutant, and VPA1514 complementary strains of *V. parahaemolyticus* under challenge by acetic acid for 1.5 hours. Gene expression was assessed by RT–PCR, and the PCR products were visualized via agarose gel electrophoresis. (A) Expression of the VPA1514 gene; (B) Expression of 16S rRNA gene as an internal control. Lane M: molecular size marker; Lane 1: wild-type strain (0 h); Lane 2: ΔVPA1514 mutant strain (0 h); Lane 3: VPA1514 complementary strain (0 h); Lane 4: wild-type strain (1.5 h); Lane 5: ΔVPA1514 mutant strain (1.5 h); Lane 6: VPA1514 complementary strain (1.5 h).

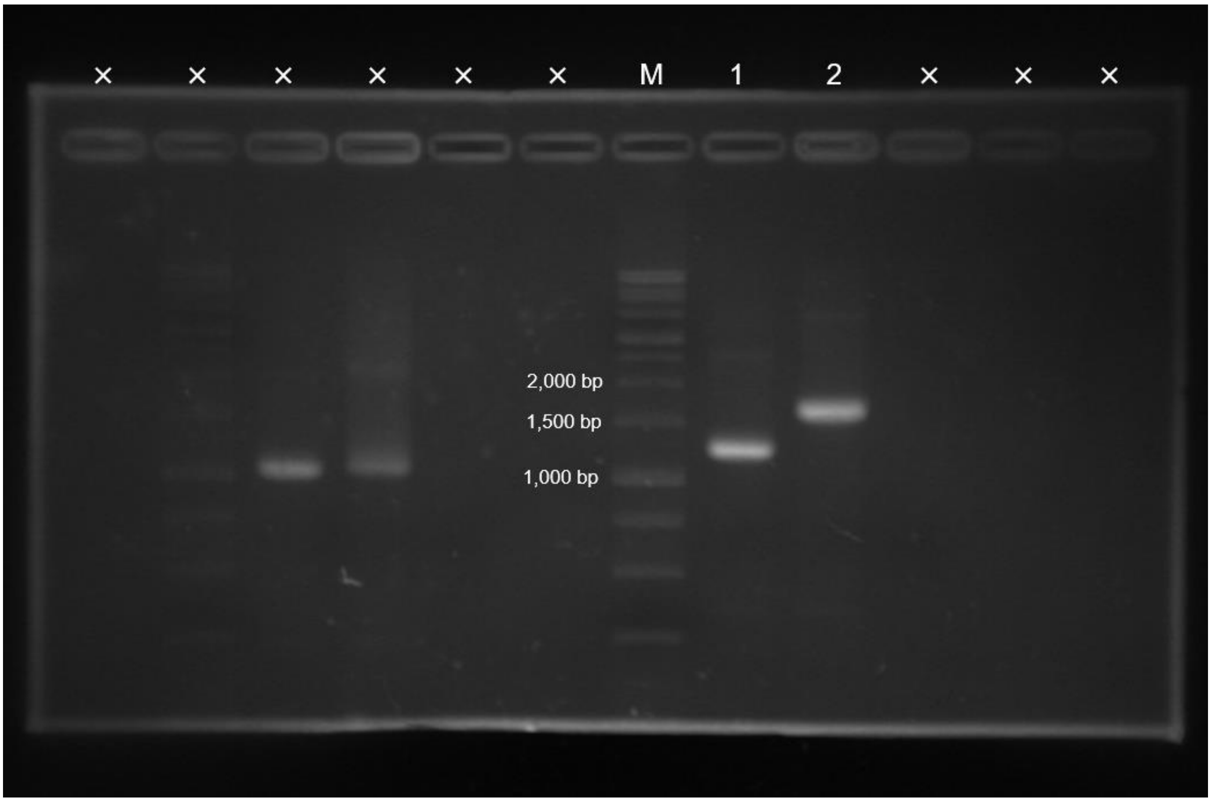


**Fig S1B original image.** Agarose gel electrophoresis of PCR products obtained from the wild-type and ΔVPA1514 mutant strains of *Vibrio parahaemolyticus*. Lane M: molecular size marker; Lane 1: ΔVPA1514 mutant strain; Lane 2: wild-type strain.

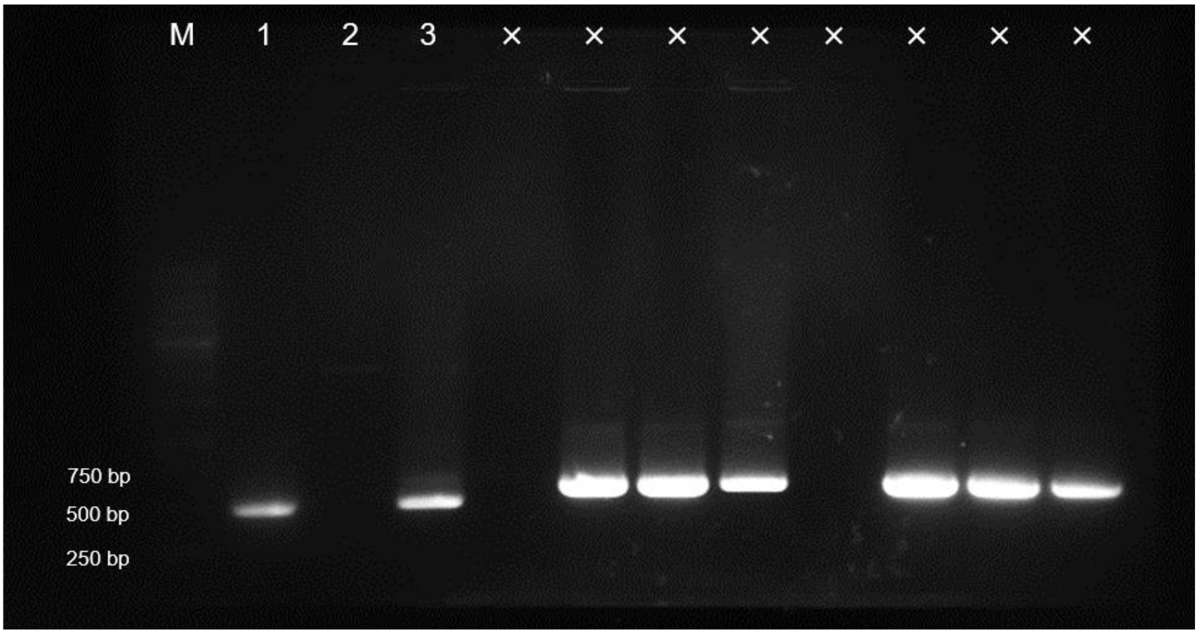


**Fig S1C original image.** Agarose gel electrophoresis of PCR products amplified from the wild-type, ΔVPA1514 mutant, and VPA1514 complementary strains of *V. parahaemolyticus*. Lane M: molecular size marker; Lane 1: wild-type strain; Lane 2: ΔVPA1514 mutant strain; Lane 3: VPA1514 complementary strain.

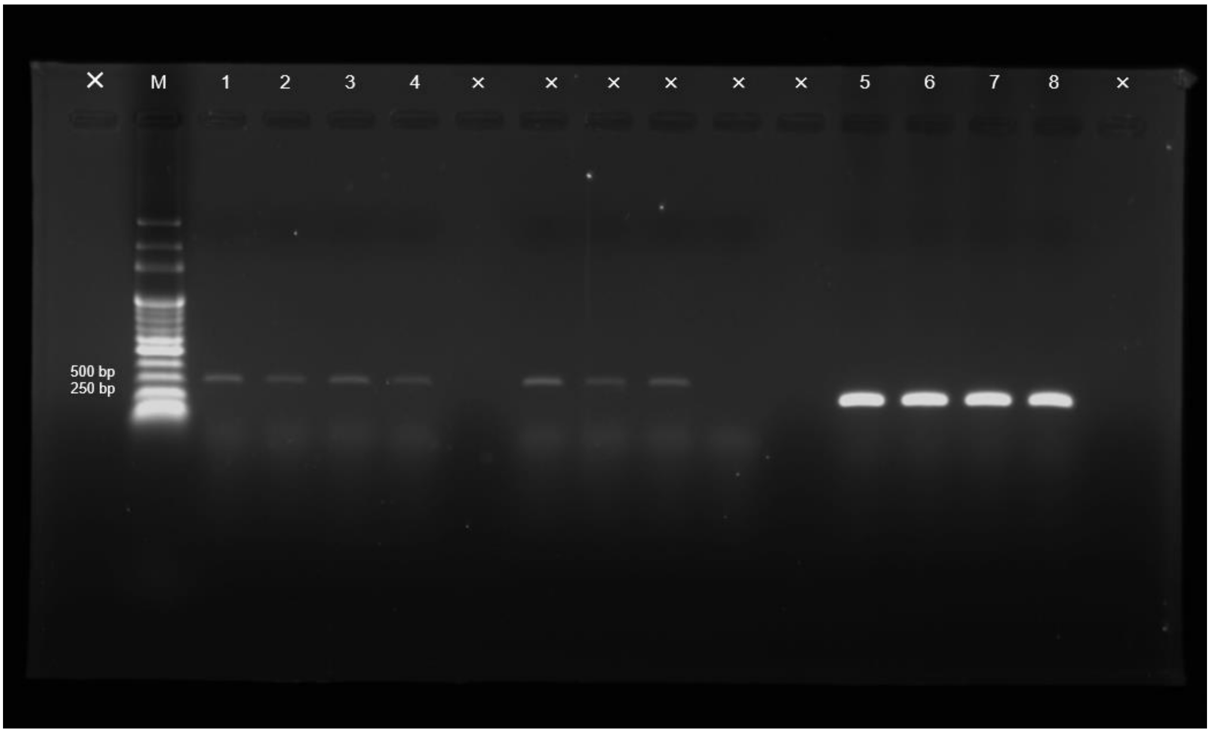


**Fig S5 original image.** RT–PCR analysis of VPA1514 gene expression in the wild-type *V. parahaemolyticus* strain during exponential and stationary phases after treatment with 30 mM acetic acid for 1.5 hours. Cultures were grown in LB- 3% NaCl at 37°C to reach either the exponential or stationary phase, then challenged with acetic acid. VPA1514 and 16S rRNA (control) expression were assessed by agarose gel electrophoresis. Lane M: molecular size marker. Lanes 1 to 4 show VPA1514 gene expression under the conditions: lane 1, exponential phase (0 h); lane 2, exponential phase (1.5 h); lane 3, stationary phase (0 h); lane 4, stationary phase (1.5 h). Lanes 5 to 8 display 16S rRNA gene expression: lane 5, exponential phase (0 h); lane 6, exponential phase (1.5 h); lane 7, stationary phase (0 h); lane 8, stationary phase (1.5 h).
